# How molecular advances may improve the diagnosis and management of PTCL patients

**DOI:** 10.3389/fonc.2023.1202964

**Published:** 2023-06-23

**Authors:** Fanny Drieux, François Lemonnier, Philippe Gaulard

**Affiliations:** ^1^ Service d’Anatomie et de Cytologie Pathologiques, INSERM U1245, Centre Henri Becquerel, Rouen, France; ^2^ Unité hémopathies Lymphoïdes, Hôpitaux Universitaires Henri Mondor, Assistance Publique des Hôpitaux de Paris, Créteil, France; ^3^ Institut Mondor de Recherche Biomédicale, INSERM U955, Université Paris Est Créteil, Créteil, France; ^4^ Département de Pathologie, Hôpitaux Universitaires Henri Mondor, Assistance Publique des Hôpitaux de Paris, Créteil, France

**Keywords:** peripheral T-cell lymphoma, molecular diagnosis, oncogenesis, diagnosis, targeted therapy

## Abstract

Peripheral T-cell lymphomas (PTCL) comprised more than 30 rare heterogeneous entities, representing 10 to 15% of adult non-Hodgkin lymphomas. Although their diagnosis is still mainly based on clinical, pathological, and phenotypic features, molecular studies have allowed for a better understanding of the oncogenic mechanisms involved and the refinement of many PTCL entities in the recently updated classifications. The prognosis remains poor for most entities (5-year overall survival < 30%), with current conventional therapies based on anthracyclin-based polychemotherapy regimen, despite many years of clinical trials. The recent use of new targeted therapies appears to be promising for relapsed/refractory patients, such as demethylating agents in T-follicular helper (TFH) PTCL. However further studies are needed to evaluate the proper combination of these drugs in the setting of front-line therapy. In this review, we will summarize the oncogenic events for the main PTCL entities and report the molecular targets that have led to the development of new therapies. We will also discuss the development of innovative high throughput technologies that aid the routine workflow for the histopathological diagnosis and management of PTCL patients.

## 1 Introduction

Peripheral T-cell lymphomas (PTCL) represent 10 to 15% of adult non-Hodgkin lymphomas. In the latest revised WHO and ICC classifications (1, 2), more than 30 entities are described, mostly defined by their clinical and pathological and phenotypic features, with a growing element of molecular data. Indeed, molecular studies based on high-throughput technologies have allowed for a better understanding of the oncogenic mechanisms involved and have improved the characterization of several entities. Although only a few specific genomic alterations define a given entity, the use of molecular data, such as clonality assays and targeted next-generation sequencing (NGS), is now integrated into the routine diagnostic workflow of expert centers, in combination with clinical and pathological clues. However, the translation of high-throughput genomic studies to clinical practice is still limited due to the high cost of high-throughput technologies and little clinical relevance for most findings. In this review, we will detail the oncogenic mechanisms of the main non-cutaneous PTCL entities, the molecular targets that have an impact on their diagnosis or treatment, and the assays that are useful for the detection of these clinically relevant molecular alterations (3). Entities with a leukemic presentation (notably T-cell large granular lymphocytic leukemia and T-prolymphocytic leukemia) will not be detailed.

## 2 Biology of PTCLs

### 2.1 Oncogenic mechanisms

**Figure 1:**
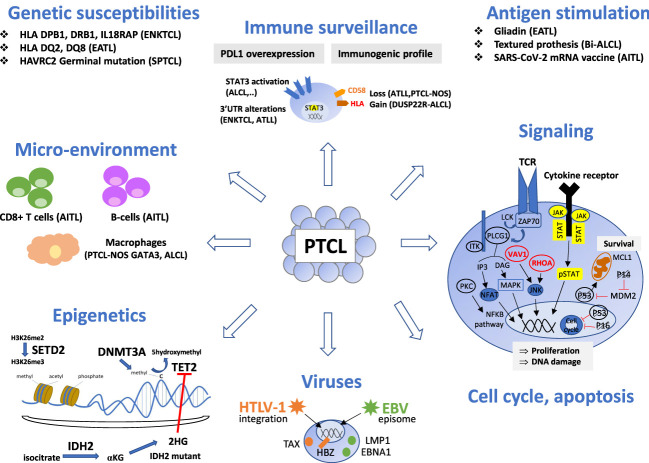
Oncogenic mechanisms of the main non-cutaneous PTCL entities. PTCL oncogenesis is a multistep process resulting from the accumulation of oncogenic events targeting epigenetics, signaling pathways (alterations of the TCR pathway is a common feature of TFH-PTCL, ATLL and certain PTCL-NOS, whereas alterations of the JAK/STAT pathway is shared by PTCL entities with a cytotoxic immunophenotype), cell cycle or apoptosis. Oncogenic viruses (HTLV1, EBV) are involved in a few specific entities. Chronic antigen stimulation may play a role as initiating event in several extranodal T or NK-cell lymphomas. Immune surveillance and crosstalk between neoplastic cells and reactive cells of the microenvironment is important, especially in AITL, where reactive cytotoxic CD8 T-cells and B-cells are associated with a poor and favorable outcome respectively. Genetic susceptibility is recognized in SPTCL, EATL and ENKTL. This figure depicts these events and their involvement for specific PTCL entities. Genes are crossed out when the alterations result in a loss of function. TFH, T follicular helper; ALCL, anaplastic large cell lymphoma; PTCL-NOS, peripheral T-cell lymphoma, not otherwise specified; ATLL, adult T-cell leukemia/lymphoma; ENKTCL, extra-nodal NK/T-cell lymphoma; HSTL, hepatosplenic T-cell lymphoma; EATL, enteropathy associated T-cell lymphoma; MEITL, monomorphic epitheliotropic intestinal T-cell lymphoma; SPTCL, subcutaneous panniculitis-like T cell lymphomas.

T-cell lymphomagenesis is a multistep process resulting from the accumulation of oncogenic events, such as genomic and epigenetic alterations and dysregulation of cellular signaling pathways, cell cycle, and immune surveillance (**Figure 1**). The microenvironment also plays a role in the initiation and maintenance of neoplastic transformation, best highlighted in angioimmunoblastic T-cell lymphoma (AITL), a disease characterized by a prominent tumor microenvironment (TME). However, the impact of the TME in other entities is still poorly understood.

Different types of genomic alterations can modify a biological function. Chromosomal translocations, detected by cytogenetic methods (karyotype, FISH, CGH), may produce fusion transcripts, detected by various technologies such as RT-PCR, RNAseq, or ld-RTPCR. They can result in aberrant expression, detectable by immunohistochemistry (for *ALK* fusions), or constitutive activation of oncogenes (such as *JAK2*, *VAV1*, *CD28*, etc.). Mutations in coding regions (single nucleotide variations or indels), detected by targeted exome or genomic sequencing, result in the gain of function of oncogenes or the loss of function of tumor suppressor genes. Mutations in noncoding regions have also been described, but their functional consequences are unclear. Disruption of the 3’UTR of PDL1 leads to its aberrant expression in extra-nodal NK/T-cell lymphomas and nasal-type (ENKTCL) and adult T-cell leukemia/lymphoma (ATLL), thus participating in immune escape (4, 5).

Epigenetic alterations appear to be a founding event in many PTCLs, mutations of genes involved in epigenetic regulation being frequently reported among different PTCL entities. Alterations of *TET2* and *DNMT3A*, reflecting clonal hematopoiesis (6), were initially described in tumoral and reactive cells of TFH lymphomas (7, 8), but have also been reported in other entities, such as peripheral T-cell lymphoma not otherwise specified (PTCL-NOS), especially with a cytotoxic immunophenotype (9), or chronic lymphoproliferative disorders of NK cells (10). Although mutations of these two genes are not sufficient to induce lymphomas (11, 12), the loss of *TET2* is often required *in vitro* and *in vivo* prior to the occurrence of other genomic alterations (such as *RHOA* G17V mutation, less frequently VAV1 alterations or *FYN*_*TRAF3IP2* fusion) as a “second-hit” in the development of TFH-lymphoma (13–15). Recurrent mutations of *IDH2* R172, responsible for the production of the oncometabolite D-2 hydroxyglutarate, measurable in the serum of patients, are confined to tumoral T-cells in AITL (16). Mutations of *TET2*, *DNMT3A* and/or *IDH2* may explain the common loss of 5-hydroxymethylcytosine observed by immunohistochemistry in most PTCL entities, with the exception of hepatosplenic T-cell lymphoma (HSTL) (17), although it has been reported independently of the mutational status. Alterations of *SETD2* that inactivate histone methyltransferase function are almost ubiquitous in monomorphic epitheliotropic T-cell lymphoma (MEITL) and less frequent in HSTL (18, 19). Mutations of several other epigenetic modifiers (*KMT2C*, *KMT2D*, *CREBBP*, *EP300*) have also been reported among the main PTCL entities (20–22).

T-cell lymphomagenesis also implies the deregulation of signaling pathways, which occurs in many PTCL entities. Dysregulation of the TCR pathway is a common feature of TFH-lymphoma, ATLL, and PTCL-NOS (23, 24), whereas the JAK/STAT pathway is frequently altered in PTCL with a cytotoxic immunophenotype (ALK-positive or negative anaplastic large cell lymphoma (ALCL), breast implant associated-ALCL (Bi-ALCL), cytotoxic PTCL-NOS, extra-nodal NK/T-cell lymphoma, nasal-type (ENKTCL), enteropathy-associated T-cell lymphoma (EATL) and MEITL (9, 18, 22, 25, 26).

Dysregulation of the cell cycle in cancer is mostly due to inactivation of the tumor suppressor gene *TP53*, which is associated with a poor prognosis. In PTCL, alterations of *TP53* and *CDKN2A*/*PTEN* have been reported in GATA3-positive PTCL-NOS, associated with complex chromosomal rearrangements and genomic instability (27–29), as well as in ENKTCL (30) and EATL (31). On the contrary, these alterations appear to be infrequent in TFH-lymphoma and ATLL (24, 29). *TP63* rearrangements, described in a small subset of ALK-negative ALCL, appear to correlate with a poor prognosis (32, 33).

Another mechanism involved in T-cell lymphomagenesis is immune escape. Overexpression of PD-L1, due to alterations in the 3’-UTR region, lead to the anergy of reactive intra-tumoral lymphocytes in ENKTCL and ATLL (4, 5). PD-L1 expression has also been described in ALK-positive and ALK-negative ALCL, regulated by STAT3 activation, with a debated impact on the prognosis (34–36). The loss of CD58, HLA molecules, or β2-microglobulin, observed in ATLL and PTCL NOS, impairs recognition of the tumor cells by the immune system (24, 28). By contrast, DUSP22-rearranged ALK-negative ALCL shows immunogenic cues, with overexpression of the genes of T-cell co-stimulation *CD58* and *CD70* and HLA class II and decreasing PDL1 expression (37).

The role of reactive immune cells and stromal cells has been highlighted in AITL, a disease in which tumor cells are commonly scarce within a prominent microenvironment, thus influencing the results of gene expression studies (38). Microenvironmental molecular signatures may have prognostic relevance: a B-cell signature is associated with a favorable outcome, whereas macrophage and CD8^+^ cytotoxic signatures correlate with an adverse prognosis (38–40). The presence of tumor-associated macrophages has also been reported to be associated with a poor prognosis in other PTCL entities, such as GATA3 PTCL-NOS (41), and ALK-positive anaplastic large cell lymphoma (ALCL) (42).

Viral infection (EBV and HTLV-1) is also recognized as a driver of PTCL oncogenesis.

- HTLV-1 infection is required for the development of ATLL. This retrovirus is randomly integrated into the host DNA (43, 44), with a predilection for specific transcription factor binding sites, such as STAT1, HDAC6, and TP53 (45). While most HTLV-1 carriers are asymptomatic, with multiple clones, a dominant clone is detected in ATLL patients (46, 47). Viral replication is permitted by clonal expansion of infected CD4^+^ T-cells (48). Expression of the oncogenic viral proteins TAX and HBZ leads to the disruption of homeostasis of infected cells, with the modification of epigenetic processes, genetic instability, and the accumulation of mutations (49). The TAX protein is highly immunogenic and responsible for the initiation of oncogenesis through NFKB and AP-1, while HBZ is involved in tumoral maintenance (50, 51).
- EBV infection is a pre-requisite for the development of ENKTCL and other NK/T-cell neoplasms, such as aggressive NK-cell leukemia or the rare EBV^+^ T/NK lymphoproliferative disorders of childhood. The mechanism for acquisition of the EBV receptor CD21 by NK and T-cells is still debated between trogocytosis and viral episome transfer (52, 53). The survival of infected cells is permitted by the type II latency pattern, with the expression of LMP1 and EBNA1 but not EBNA2. LMP1 promotes the proliferation of EBV-infected cells through deregulation of the p53, CMYC, and NF-κB pathways, in synergy with the production of cytokines (IL-2, IL-9, IL-10 et IL-15), by infected neoplastic cells and cells of the microenvironment (54).

Antigenic stimulation may also play a role in the initiation or progression of T/NK cell lymphomagenesis, as established for gliadin in EATL (55), textured breast-implants in Bi-ALCL (56), or recently suggested for the SARS-CoV-2 mRNA vaccine in AITL (57).

Finally, genetic susceptibility has been identified in several entities, notably association between the haplotypes HLA-DPB1, HLA-DRB1, and IL18RAP and ENKTCL (58, 59), HLA DQ2/DQ8 and EATL (60), and germline mutations of *HAVRC2* in subcutaneous panniculitis-like T-cell lymphoma (61).

### 2.2 Oncogenic events of the main non-cutaneous PTCL entities

PTCL can be derived from cells of the innate or adaptative immune system. Neoplasms likely deriving from the innate immune system comprise mostly extra-nodal lymphomas (ENKTCL, EATL, MEITL, HSTL, γδ-lymphomas, and probably cases among PTCL-NOS). They share a cytotoxic phenotype, alterations of the JAK/STAT pathway, and a context suggestive of chronic antigen stimulation. PTCL derived from cells of the adaptative immune system include most lymphomas with a nodal presentation with a T helper phenotype, such as TFH-lymphomas, ATLL, and PTCL-NOS. These lymphomas often show dysregulation of the TCR signaling pathway, in addition to alterations of epigenetic modifiers. The molecular characteristics of the main non cutaneous PTCL entities are summarized in **Table 1**.

**Table 1:** Molecular characterization of the main non-cutaneous PTCL entities.

Entity	Differentiation	Molecular features
TFH-lymphomas	TFH	- DNA methylation: *TET2*, *DNMT3A*, *IDH2* R172 mutations - TCR pathway: *RHOA* G17V, *CD28*, *VAV1*, *PLCG1* mutations *-* Fusion transcripts: *ICOS*_*CD28*, *CTLA4*_*CD28*, *ITK*_*SYK*, *ITK*_*FER*, fusion transcripts involving *VAV1*
ALK-positive ALCL	Activated cytotoxic T cell	- Fusion transcripts involving *ALK* - Mutations in genes of the NOTCH1 pathway
ALK-negative ALCL	Activated cytotoxic T cell	STAT3 activation: *JAK1* and/or *STAT3* mutations Fusion transcripts involving *ROS*, *TYK2*, *FRK, CAPRIN2* Absence of STAT3 activation: *DUSP22*/*IRF4* (locus 6p25.3) rearrangement *MSC*E116K mutation Others: *TP63* rearrangements
Breast-implant ALCL	Activated cytotoxic T cell	- JAK/STAT pathway: *STAT3*, *JAK1*, *SOCS3*, *STAT5B*, *SOCS1*, *PTPN1* mutations - Epigenetics: *KMT2D*, *KMT2C*, *CREBBP*, *CHD2, TET2, DNMT3A* mutations
ATLL	Memory regulator T cell	*-* TCR pathway: *PLCG1, PRKCB, CARD11, VAV1, IRF4, FYN, CCR4, CCR7, RHOA, CD28* mutations - Immunosurveillance: *CD58, B2M, HLA* (class I) mutations *-* JAK/STAT pathway: *JAK3, STAT3, PTPN1* mutations *-* Transcription factor: *GATA3, IKZF2, PRDM1* mutations - Epigenetics: *TET2, DNMT3A, IDH2, SETD2, EP300, KDM6A* mutations - Fusion transcripts: *ICOS_CD28* and/or *CTLA4_CD28*
ENKTCL (nasal type)	NK>>T (γδ or αβ)	- *BCOR*, *DDX3X*, *TP53*, *MGA*, *STAT3*, *STAT5B*, *MLL2*, *ARID1A*, *MSN* mutations - 3 molecular subgroups : °TSIM : mutations of genes of the JAK/STAT pathway, *TP53*, amp9p24.1(*JAK2*, *PDL1/2*), amp17q21.2 (*STAT3*/*5A*/*5B*), EBV latency type II => NK cells °HEA: mutations of *HDAC9*, *EP300*, *ARID1A*, EBV latency type II => T-cells °MB: mutations of *MGA*, del1p22.1 (*BRDT*), *MYC* overexpression, EBV latency type I => T-cells
HSTL	Tgδ> Tαβ	*- SETD2, STAT5B, INO80, ARID1B, STAT3, PIK3CD* mutations
Indolent clonal T-cell lymphoproliferative disorder of the gastro-intestinal tract	CD8+ or CD4-/CD8- (TH2)	- Structural alterations of the 3’UTR regions of IL2 coding gene
	CD4+ or CD4+/CD8+	- JAK/STAT pathway: *STAT3*, *SOCS1* mutations, *STAT3*_*JAK2* fusion - Epigenetics: *TET2*, *DNMT3A*, *KMT2D* mutations
EATL	Intraepithelial lymphocyte (Tαβ)	*-* JAK/STAT pathway: *JAK1* (p.G1097 dans 50%), *JAK3*, *STAT3*, *STAT5B*, *SOCS1* mutations - *KRAS*, *NRAS* mutations - NFκB pathway: *TNFAIP3*, *TNIP3* mutations - Epigenetics: *TET2*, *KMT2D*, *DDX3X*, *SETD2* (15%) mutations
MEITL	Intraepithelial lymphocyte (Tγδ > Tαβ)	- Alterations of *SETD2* (mutations, deletions) - *STAT5B*, *JAK3*, *TP53*, *GNAI2* mutations
T-LGLL	Tαβ (CD8) >> Tγδ	*-* JAK/STAT pathway: *STAT3*, less frequently *STAT5B* mutations - Epigenetics: *TET2*, *DNMT3A*
PTCL-NOS	TH1 (αβ >> δγ), common cytotoxic phenotype	- Epigenetic: *TET2*, *DNMT3A*, *KMT2D*, *SETD2* mutations - TCR pathway: *VAV1*, *PLCG1*, *PRKCB*, *CARD11* mutations*;* fusion transcripts involving *VAV1* - JAK/STAT pathway: *STAT3*, *STAT5B*, *JAK3*, *SOCS1* mutations
	TH2 (Tαβ)	- Deletions of *CDKN2A*, *TP53*, *PDGFA*, *STK11*, *WDR24*, *CDK4*, *CCND1*, *AKT*, *RPTOR*… - Gains/amplifications of *STAT3*, *CMYC*

TFH, T follicular helper; ALCL, anaplastic large cell lymphoma; PTCL-NOS, peripheral T-cell lymphoma, not otherwise specified; ATLL, adult T-cell leukemia/lymphoma; ENKTCL: extra-nodal NK/T-cell lymphoma, HSTL, hepatosplenic T-cell lymphoma; EATL, enteropathy associated T-cell lymphoma; MEITL, monomorphic epitheliotropic intestinal T-cell lymphoma; T-LGLL, T-cell large granular lymphocytic leukemia.

#### 2.2.1 Nodal TFH lymphomas

In the revised 2022 WHO and ICC classifications, the family of lymphomas derived from TFH cells is regarded as a single disease encompassing three morphological subtypes, commonly designated angio-immunoblastic T-cell lymphoma (AITL), follicular-type, and not otherwise specified. They have distinct morphological features but share a common TFH phenotype and signature, as well as a similar molecular pattern. In routine practice, the TFH phenotype is defined by the expression of CD4, with at least two TFH markers among PD1, ICOS, CD10, CXCL13, and BCL6, although none of them, in particular PD1 and ICOS, are fully specific, as they can be expressed by non-TFH reactive cells or other non-TFH PTCLs (62–65). TFH-lymphomas show a unique mutational landscape, characterized by the accumulation of alterations in genes involved in epigenetic regulation (*TET2*, *DNMT3A*, *IDH2*) *(* 7, 11, 66) and the TCR pathway (*RHOA*, *VAV1*, *CD28*, *PLCG1*, *FYN*, *LCK*) (23, 67–73). Fusion transcripts involving genes of the TCR signaling (*ICOS*_*CD28*, *CTLA4*_*CD28*, *ITK*_*SYK* or involving *VAV1* with multiple partners) and NFKB (*FYN*_*TRAF3IP2*) pathways can be observed. Although mutations of *TET2* and *DNMT3A* may be observed in tumoral and reactive cells, hotspot mutations in *RHOA* G17V and *IDH2* R172 are thought to be restricted to the TFH tumor cells (74, 75). The recurrent *RHOA G17V* mutation, detected in 50 to 70% of AITL (23, 67–69, 75–78), impairs the GTPase domain, showing dominant negative activity and thus abolishing GTP binding and downstream signaling. This mutation is also responsible for VAV1 phosphorylation and TCR pathway activation (71). *RHOA G17V* drives TFH polarization and promotes lymphomagenesis *in vivo* through ICOS-PI3K-mTOR signaling (14, 15). The *IDH2* R172K mutation combined with *TET2* alterations modulate the tumoral microenvironment, promoting B-cell proliferation, the accumulation of plasma cells, and angiogenesis (79). Mutations in *CD28*, observed in 10% of TFH-PTCL, are reported to be mutually exclusive from fusion transcripts involving *CD28* and other genes of the TCR pathway (23, 72, 73, 76, 80, 81). Alterations in *VAV1* result in oncogenic activation of the NFAT pathway (70, 71, 82). Alterations of many other genes of the TCR pathway (*FYN*, *PLCG1*, *PIK3R1*, *PDPK1*, *AKT*, *LCK*, *TRAF6*) contribute to T-cell proliferation (23). The rare *ITK*_*SYK* fusion transcript has been described in follicular-type and in rare cases of AITL (83, 84). TFH lymphomas illustrate multistep oncogenesis, as shown by the development of « AITL » tumors *in vivo* in *TET2* knock-out mice transfected with a *RHOA* mutated gene (13, 14), or in double-mutant mice TET2/IDH2R172K (79).

Overall, although there is no pathognomonic genomic alteration that defines the TFH category, the detection of *RHOA G17V* and/or *IDH2 R172* mutations and, to a lesser extent, fusion transcripts involving *CD28* or *TRAF3IP2* constitute a supplemental clue to the diagnosis for pathologists.

#### 2.2.2 - Anaplastic large-cell lymphomas

This category, defined by large “hallmark” cells showing strong and homogenous CD30 expression by immunohistochemistry, includes several entities based on the association of ALK-rearrangement and the clinical presentation as systemic, cutaneous, or breast implant-associated disease. Cutaneous ALCL are not considered here.

A) ALK-positive ALCL is the only entity defined by recurrent genomic translocations involving the *ALK* gene on chromosome 2p23 with various partners, the most frequent (~80%) being *NPM1*. The translocation produces an oncogenic fusion protein consisting of the association of the N-region of a partner gene with the catalytic tyrosine kinase domain of ALK, resulting in constitutive activation by dimerization. The chimeric NPM1_ALK protein triggers several oncogenic pathways (JAK/STAT, PI3K, MAPK, PLCG), leading to neoplastic transformation (85, 86), whereas *TRAF1*_*ALK* activates the NFKB pathway (87, 88). Recently, mutations of *NOTCH1* and genes of the TCR pathway have also been reported (89). The diagnosis is based on the detection of aberrant ALK expression by immunohistochemistry using anti-ALK antibodies. The pattern of staining may be nuclear +/- nucleolar and/or cytoplasmic, depending on the partner gene involved in the translocation (**Table 2**). The disease, which mainly occurs in children and young adults, follows a generally favorable prognosis (5-year OS around 90%) after chemotherapy with CHOEP (100–102) or BV-CHP (103). The prognosis may be less favorable in cases with secondary MYC overexpression or rearrangement, in certain histologic variants (small-cell or lymphohistiocytic) occurring in children (88, 104, 105).

**Table 2:** Fusion transcripts involving *ALK* in ALK-positive anaplastic large cell lymphoma.

Fusion	Translocation	Immunostaining
***NPM1*_*ALK (*** 90)	t (2,5)(p23.2;q35.1)	Nuclear and cytoplasmic
***TPM3*_*ALK (*** 91)	t(1;2)(q25;p23)	Cytoplasmic with peripheric reinforcement
***ATIC*_*ALK (*** 92)	inv(2)(p23q35)	Cytoplasmic diffuse
***TFG*_*ALK (*** 93)	t(2;3)(p23;q12.2)	Cytoplasmic diffuse
***CLTCL*_*ALK (*** 94)	t(2;17)(p23;q23)	Cytoplasmic granular
***MSN*_*ALK (*** 95)	t(X;2)(q11-12;p23)	Membranous
***ALO17*_*ALK (*** 96)	t(2;17)(p23;q25)	Cytoplasmic diffuse
***MYH9*_*ALK (*** 97)	t(2;22)(p23;q11.2)	Cytoplasmic diffuse
***TRAF1*_*ALK (*** 87)	t(2;9)(p23;q33)	Cytoplasmic
***EEF1G*_*ALK (*** 98)	t(2;11)(p23;q12.3)	Cytoplasmic
***PABPC1_ALK (*** 99)	t(2;8)(p23;q22)	Cytoplasmic

B) Systemic ALK-negative ALCL is still heterogeneous in the current classifications, gathering cases with different oncogenic pathways:

- Rearrangement of the 6p25.3 locus involving *DUSP22* and *IRF4* (106) defines a peculiar subgroup (approximately 25-30% of ALK-negative ALCL), characterized by a non-cytotoxic phenotype, silencing of the tumor suppressor gene *DUSP22* while showing normal *IRF4* expression, absence of STAT3 activation, global DNA hypomethylation, an immunogenic molecular profile (overexpression of CD58, CTA, HLA class II), and expression of LEF1 (37, 107–110). Recurrent *MSC E116K* mutations are responsible for activation of the CD30-IRF4-CMYC axis and the dysregulation of cell cycle arrest (111). These rearrangements were initially detected by mate-pair DNA sequencing in the context of a translocation t(6,7)(p25.3;q32.3) also involving the non-coding gene *FLJ43663* at the fragile site FRA7H of chromosome 7 (106). The prognosis is debated, favorable in most studies (112, 113) but not confirmed in others (114, 115).

- Rearrangements of *TP63*, due to the inversion inv (3) (q26q28) or translocation t(3,6)(q28;p22.3) that produce the fusion transcripts *TBL1XR1*_*TP63* and *TP63*_*ATXN1* respectively, coding for oncogenic chimeric proteins, are rare and associated with a poor prognosis (32, 112, 114). The detection of P63 by immunohistochemistry may reflect P63 overexpression independently of the presence of fusion transcript (33).

- Aberrant truncated transcripts of *ERBB4* was also reported in 24% of ALK-negative ALCL in one study, associated with a Hodgkin-like morphology, without clinical relevance (116).

- Expression of pSTAT3 by immunohistochemistry, reflecting activation of the JAK/STAT pathway, is common in ALK-positive and ALK-negative ALCL, with the notable exception of those cases associated with *DUSP22* rearrangement. Among ALK-negative ALCLs, a recent study that excluded cases with rearranged *DUSP22* suggested that positive pSTAT3 cases constitute a distinct subgroup, characterized by a cytotoxic phenotype and the expression of EMA and PDL1, that is associated with a better prognosis than negative pSTAT3 cases (117). Such constitutive phosphorylation of STAT3 has been previously shown to be related to mutations in *JAK1* and/or *STAT3*, reported in 18% of ALK-negative ALCLs, as well as in fusion transcripts involving *ROS, TYK2*, and *FRK* (26, 118). More recently, fusion transcripts involving *JAK2* with several partners (*PABPC1*, *PCM1*, *ILF3*, *TFG*, *MAP7*) were detected by targeted RNAseq and associated with a Hodgkin-like morphology (119).

C) Breast-implant associated ALCL is a site-specific entity that occurs after a long latency after a breast implant for reconstruction or cosmetic reasons. Most cases are non-invasive. The disease appears to be due to chronic inflammation, with possible TH2 polarization, linked to a macro-textured implant (120). High-throughput sequencing studies have highlighted alterations of genes involved in the JAK-STAT pathway (*STAT3*, *STAT5B*, *JAK1*, *JAK3*, *SOCS1*, *SOCS3*), leading to its constitutive activation, together with recurrent mutations in epigenetic modifiers (*KMT2C*, *CREBBP*) *(* 22, 121), the loss of chromosome 20 (122), and chromosome 9p24 gains, leading to PDL1 expression (123). Recently, a *STAT3*_*JAK2* fusion transcript was also reported (124).

Several immunohistochemical algorithms have been recently proposed to classify ALCL based onLEF1, P63, and pSTAT3 (117, 125), although this currently has no impact on the management of ALK-negative ALCL patients.

#### 2.2.3 EBV-positive NK or T-cell neoplasms

EBV-related NK or T-cell neoplasms are heterogenous diseases derived from T or NK cells (126). The revised WHO and ICC classifications recognize ENKTCL, and primary nodal EBV-positive T/NK-cell lymphomas, characterized by nodal involvement, as distinct entities (1, 2). In addition to EBV, considered to be a driver of oncogenesis in these lymphomas, defined by EBV infection of virtually all neoplastic cells, as shown by *in situ* hybridization with EBER probes, the mutational landscape of ENKTCL is characterized by recurrent mutations of genes coding for RNA helicases (especially *DDX3X*), as well as *TP53*, genes of the JAK/STAT pathway (*JAK3*, *STAT5B*, *STAT3*) and epigenetic modifiers (*MLL2*, *ARID1A*, *EP300*, *ASXL3*) (20, 30, 127). The initial poor prognosis associated with *DDX3X* and *TP53* mutations for patients treated with the CHOP regimen was not confirmed for patients receiving L-asparaginase treatment (20, 30, 128). Recurrent deletions of the 6q21 locus encompassing tumor suppressor genes (*PRDM1*, *ATG5*, *AIM1*, *FOXO3* et *HACE1*) have been detected by CGH array (129–131). A recent large integrative analysis of genome, exome, and RNA sequencing, identified three molecular subgroups (30):

- the “TSIM (tumor suppressor and immunomodulator)” subgroup is characterized by frequent *TP53* mutations, deletion of the 6q21 locus, amplification of the 9p24.1 locus containing *PDL1* and *PDL2*, and the amplification of genes of the JAK/STAT pathway. This subgroup presents a gene expression signature enriched in NK-cell genes. There is an EBV latency II phenotype, with expression of the lytic gene *BALF3*, responsible for DNA damage and genomic instability.
- the “MB (MGA, BRDT)” subgroup is characterized by frequent *MGA* mutations, loss of heterozygosity of *BRDT*, and MYC overexpression, as well as activation of the MAPK, NOTCH, and WNT pathways. The EBV latency is of type I, with downregulation of *LMP1*.
-the “HEA (HDAC, EP300, ARID1A)” subgroup is characterized by mutations of epigenetic modifier genes (*HDAC9*, *EP300* et *ARID1A*), resulting in aberrant histone acetylation. The gene expression profile is enriched in T-cell genes and shows activation of the TCR and NFKB pathways. The EBV latency is of type II, with expression of the *BNRF1* lytic gene.

Although there is currently no applicability of this molecular subclassification in routine practice, the poor prognosis of the MB subgroup relative to TSIM and HEA (3-year OS rate of 38% versus 80% and 90%, respectively) may justify the evaluation of MYC expression in ENKTCL. Structural alterations of *CD274*, coding for PDL1, appear to confer sensitivity to immune checkpoint inhibitors (5, 132).

EBV-positive nodal T- and NK-cell lymphoma or primary nodal Epstein-Barr virus–positive T-cell/NK-cell lymphoma, is now recognized as a distinct entity in both the WHO and ICC classifications respectively, due to its differences with ENKTCL. This entity is morphologically characterized by the lack of necrosis and angiocentrism, a common CD8^+^ CD56^-^ phenotype, a frequent T-cell origin, and, finally, peculiar molecular abnormalities, with frequent *TET2*, *PIK3CD*, and *STAT3* mutations, activation of the NFKB, IFNγ, and JAK-STAT3 pathways, resulting in high PDL1 expression, and lower genomic instability (133). The prognosis is reported to be poorer than for ENKTCL.

The mutational landscape of ENKTCL is shared with that of other EBV-positive NK/T-cell neoplasms, in particular, aggressive NK-cell leukemia (134–136), as well as that of chronic active EBV-disease (137). This genetic landscape may be of clinical relevance in the rare cases that require a differential diagnosis from infectious mononucleosis.

#### 2.2.4 Adult T-cell Leukemia/Lymphoma

This HTLV-1-associated T-cell neoplasm occurs after a long latency (more than 25-30 years) following infection, mainly due to prolonged breast feeding and, less frequently, sexual transmission (138). The histopathological diagnosis is challenging in the absence of information concerning the HTLV-1 status, as the pathological aspects of ATLL are highly heterogeneous. It can be evoked by the loss of CD7, together with the expression of CD25 and FOXP3, although the CD25^+^/FOXP3^+^ immunophenotype is variable and not specific to ATLL (139–142). The molecular landscape is characterized by mutations in genes of the TCR pathway (*PLCG1*, *PRKCB*, *CARD11*, *VAV1*, *IRF4*, *FYN*, *CCR4*, *CCR7*, *RHOA*, *CD28*), JAK/STAT pathway (*JAK3*, *STAT3*, *PTPN1*), immune surveillance (*CD58*, *B2M*, *HLA* class I), DNA damage (*TP53*, *CDKN2A*, *POT1*), epigenetic modifiers (*TET2*, *DNMT3A*, *IDH2*, *SETD2*, *EP300*, *KDM6A*), transcription factors (*GATA3*, *IKZF2*, *PRDM1*), and fusion transcripts involving CD28 (*ICOS*_*CD28* and *CTLA4*_*CD28*) *(* 24, 143). The co-expression of these two fusion transcripts can occur in patients younger than 50 years of age (144). Gene mutations of the TCR/NFKB pathway, *TP53*, and *IRF4* are associated with an aggressive outcome, whereas *STAT3* mutations are frequently observed in patients with more indolent disease (143, 145). The type of *CCR4* mutation also has a specific prognostic impact (unfavorable in cases of frameshifts vs non-synonymous variations) (146).

#### 2.2.5 Intestinal T-cell lymphomas

Enteropathy-associated T-cell lymphoma (EATL) and monomorphic epitheliotropic intestinal T-cell lymphoma (MEITL) are two distinct entities, with different morphological and immunophenotypic features. Although both are derived from intestinal intra-epithelial lymphocytes (IEL) expressing CD103, EATL and MEITL show distinct clinico-pathological and molecular characteristics (31).

- EATL is associated with celiac disease or gluten sensitivity. Its histopathological features include the proliferation of pleomorphic to anaplastic T-cells expressing CD3 and CD30, but lacking CD4 and CD8, despite an activated cytotoxic profile. CD103 is variably expressed. Overexpression of P53 is detectable by immunohistochemistry, independently of gene alterations (147). This entity shows frequent alterations of the JAK/STAT pathway (in particular, *STAT3* and *JAK1*, as well as *SOCS1* and *SOCS3*), whereas *STAT5B* mutations are almost constantlyabsent (18, 148, 149). *TET2* and, less frequently, mutations of the RAS/MAPK pathway (149) can be observed, whereas *SETD2* mutations were almost absent in most recent series (18, 148). Gene expression profiling studies have shown enrichment for genes of the JAK/STAT (*STAT3*, *STAT5A*) and IFNγ pathways (31).
- MEITL does not associate with celiac disease and is typically characterized by the proliferation of monomorphic medium cells, showing epitheliotropism and a CD8^+^ CD56^+^ phenotype. However, approximately 25% of cases may show more pleomorphism and certain phenotypic variations associated with the prognosis, in particular, a better outcome in the presence of aberrant expression of CD20 or poor outcome in the presence of *MYC* expression and *TP53* alterations, suggesting the utility of screening for these abnormalities in routine practice (150, 151). MEITL has a very homogeneous genetic landscape, with almost consistent alterations of *SETD2* (mutation +/- deletion) associated with mutations of *STAT5B* (approximately 60%) or *JAK3* and *GNAI2*, which constitute a common feature and may help pathologists in difficult cases (18, 150–152).

Indolent clonal T-cell lymphoproliferative disorder of the gastrointestinal tract (2), also designated indolent T-cell lymphoma of the gastrointestinal tract in the WHO classification (1), is now recognized as a definitive entity in both classifications due to the recent evidence of neoplastic molecular features, i.e., alterations of genes in the JAK/STAT pathway or epigenetic modifier genes and *JAK2*_*STAT3* fusions or structural alterations of the 3’UTR of the IL2 gene, depending on the CD4 or CD8 phenotype (153, 154). Despite an indolent course, some cases may relapse, spread to other sites, or transform, indicating potential aggressiveness (155, 156).

Indolent NK-cell lymphoproliferative disorder of the gastrointestinal tract is a rare condition, and a new entity in the WHO and ICC classification. Although neoplastic molecular characteristics have also been described, in particular, recurrent deletions of *STAT3*, there is no extension of this lymphoproliferation beyond the gastrointestinal tract and the outcome is favorable (157).

#### 2.2.6 Hepatosplenic T-cell lymphoma

This rare neoplasm occurs preferentially in young males but can arise at any age, with a possible context of immunosuppression. The diagnosis is based on highly characteristic pathological features, in particular sinus infiltration in the bone marrow by small to medium lymphocytes with a CD3^+^, CD5^+^, CD4^-^/CD8^-^, CD56^+^ phenotype, commonly TCRγδ^+^. The sinusal infiltration in the liver and spleen is less specific. There is typically no lymph node involvement. This entity was initially characterized by an isochromosome 7q and chromosome 8 trisomy (158, 159), but cytogenetic material is not always available in routine practice to support the diagnosis and FISH analysis can be challenging. The mutational landscape has been reported, identifying three types of mutations involving 1/epigenetic modifier genes (*SETD2*, *ARID1B*, *INO80*, *TET3* and *SMARCA2*), 2/*STAT5B* or *STAT3* that are mutually exclusive, and 3/*PIK3CD* (19, 160, 161). Gene expression profiling studies show a distinct signature, characterized by the overexpression of oncogenes (*FOS*, *FOSB VAV3, MAF)*, NK-cell associated genes (*KIR3DS1*, CD244 and other KIRs), the tyrosine kinase *SYK*, and *S1PR5*, and downregulation of *AIM1*, which could constitute targets for therapy in this disease that has always fatal outcome (162). A recent single-cell profiling study suggested a change in the gene expression profile of the tumor cells during disease progression under the selective pressure of therapy (163).

#### 2.2.7 PTCL-NOS

PTCL-NOS is a diagnosis of exclusion, corresponding to cases that do not fulfill the criteria for defined PTCL entities. Thus, a large panel of immunohistochemical markers and the integration of clinical and often molecular features are required to exclude any other PTCL. Gene expression profiling studies have shown two subgroups based on expression of the TBX21 and GATA3 transcription factors associated with the immunological TH1 and TH2 signatures, respectively (39, 40), confirmed by immunohistochemistry (164). The TBX21 group is enriched in genes of IFNγ and NFKB pathway signatures and shows mutations of genes involved in epigenetic regulation (*TET1*, *TET3*, *DNMT3A*), whereas the GATA3 group shows a cell proliferation signature driven by MYC, together with enrichment in PI3K/Akt/mTOR pathway signatures, a higher number of genomic copy number abnormalities, and a poorer outcome (27, 165). In routine practice, there is no consensus concerning the proposed thresholds of immunohistochemical markers to define these two subgroups and an understanding of the clinical relevance of such immunohistochemical algorithms requires further studies.

The mutational landscape of PTCL-NOS is currently poorly defined, likely due to the heterogeneity of this category. Only a few “omic” studies focusing on PTCL-NOS have been published to date. Targeted sequencing has shown mutations of epigenetic modulator genes, notably histone methylation (*KMT2D*, *SETD2*, *KMT2A*, *KDM6A*) or acetylation (*EP300*, *CREBBP*), as well as that of genes of the TCR pathway (*TNFAIP3*, *TRAF3*, *TNFRSF14*) and tumor suppressor genes (*TP53*, *ATM*, *FOXO1*, *BCORL1*) *(* 21, 166). Recent integrative studies based on exome and RNA sequencing have confirmed mutations of genes involved in epigenetic regulation (*TET2*, *DNMT3A*, *KMT2C*, *KMT2D*, *SETD2*, *CREBBP, ARID1A*), tumor suppressor genes (*TP53*, *TP63*, *ATM*, *FAT1*, *LATS1*, *STK3*), and genes of the NOTCH pathway (NOTCH1 and 2) (28, 167). In one study, mutations in *FAT1* were shown to be associated with a poor prognosis (167). RNAseq studies have shown fusion transcripts involving *VAV1* with various partner genes (*GSS*, *THAP4*, *MYO1F*, *S100*, *HNRNPM*) and rearrangements of *VAV1* were detected by FISH in 11% of PTCL-NOS (28, 70, 82). The *VAV1*_*MYO1F* transcript induces tumoral TH2 polarization and the accumulation of tumor-associated macrophages (41). Other fusion transcripts have also been reported in single cases (*ITK*_*FER*, *IKZF2*_*ERBB4*, *ETV6*_*FGFR3*) *(* 82). A t (14, 19)(q11;q13) translocation, involving *TCRA* and the poliovirus receptor-related 2 gene (*PVRL2*), resulting in *BCL3* overexpression, has also been reported in PTCL-NOS, including one case with the morphological variant of Lennert’s lymphoma (168, 169).

PTCL-NOS with a cytotoxic phenotype has been reported in 25 to 40% of cases, associated with impaired immunity and a poor prognosis (9, 170). This immunophenotypic subgroup has also been identified in gene expression studies within the PTCL-NOS TBX21 subgroup, enriched for genes of CD8/NK cells, the IFN response, and an immunosuppressive signature (39, 40). Targeted sequencing has shown recurrent mutations of genes involved in epigenetic regulation (*TET2*, *DNMT3A*), TCR (*VAV1*, *PLCG1*, *PRKCB*, *CARD11*) and the JAK/STAT pathways, as well as *TP53 (* 9). Fusion transcripts involving *VAV1* have been detected in 14% of patients. In another study, two cases of cytotoxic PTCL-NOS with diffuse cutaneous and medullary involvement showed a t (6, 14)(p25;q11.2) translocation resulting from rearrangement between the TCRα and IRF4 loci (171).

Despite these advances in our knowledge of the molecular biology of this entity, there is still an unmet need for the management of PTCL-NOS patients.

## 3 From biology to the diagnosis and management of PTCL patients

The diagnosis and classification of PTCLs are often challenging for pathologists, requiring experienced hematopathologists and access to molecular tests. In the absence of clear diagnostic guidelines, practices are often heterogenous between centers (172–174).

Analysis of rearrangements of the TCR loci (especially *TRG* or *TRB*) is an important element of the diagnostic process. PCR-based assays (BIOMED-2) are largely widespread in routine practice due to their reliability on FFPE samples (160–162). However, there are a number of pitfalls in the interpretation of clonality testing due to “false-negative” results in cases with low tumoral content, especially common in AITL, or due to T-cell oligoclones, as observed in AITL (175). Conversely, the presence of clonal TCR rearrangements in certain reactive conditions or even in B-cell lymphomas (notably Hodgkin lymphomas) due to TCR repertoire restriction can be misleading (176). The development of NGS-amplicon based clonality assays may improve the detection of scarce clones in a polyclonal background and allow the determination of clonotypes (177). Several authors have proposed analyzing TCR genes by whole genome sequencing, but its applicability in routine practice is still limited (178). Others have highlighted the potential interest of analyzing non-recombined T-cell receptor sequences using a digital PCR assay (179).

Recently, gene expression studies suggested molecular classifiers to discriminate the main PTCL entities, with certain limitations due to tumor cell content and the quality of the nucleic acid (40, 180, 181). Such tools should be used in routine practice with caution, as they were developed for the classification of the most common entities, their robustness has not yet been extensively evaluated, and the results need to be interpreted in the context of the histopathological analysis. Indeed, misclassification using these algorithms or discordance with the histopathological data occur for 15 to 20% of samples, likely due to a prominent microenvironment or plasticity of the tumor cells (180, 181). Sequencing of transposase-accessible chromatin (ATAC-seq) has been proposed as another innovative strategy to classify PTCL (182), but it requires fresh or frozen samples and its applicability in routine practice has not been yet evaluated.

Exome and genome sequencing studies have allowed a precise description of the mutational landscape of almost all PTCL entities. An increasing number of laboratories have developed targeted NGS panels for the molecular characterization of lymphomas or hematological neoplasms that are useful for their diagnosis and classification (183). The diagnostic performance of targeted NGS relative to that of measuring T-cell clonality by BIOMED multiplex PCR in PTCL was assessed in one study and showed similar sensitivity (approximately 95%) but significantly superior specificity (100% versus 45%) (184). However, there is currently no consensus concerning the design of the panel or the sequencing depth or coverage, which may affect the interpretation of the results. Hotspot mutations of diagnostic relevance, notably *RHOA G17V* or *IDH2 R172* mutations, can also be detected using alternative technologies, such as allele-specific PCR, digital PCR, and RTMLPA (180, 185–188).

As described above, despite highly characteristic genetic profiles for certain PTCLs, such as TFH-lymphomas and MEITL, there is no single pathognomonic molecular alteration that can define an entity, apart from ALK-positive ALCL. However, the detection of *RHOA* G17V and *IDH2* R172 mutations in routine practice strongly supports the diagnosis of TFH-PTCL (14, 16, 63, 64, 72). Although *RHOA* mutations can also be observed in 10% of ATLL, only 1% correspond to G17V (189), whereas *IDH2* R172 is almost specific to AITL.

Within ALCL, the discovery of the translocation t(2,5) led to the development and use of an anti-ALK antibody in routine practice, allowing rapid and efficient determination of the ALK status by immunohistochemistry (90, 190). The identity of the *ALK* gene partner does not appear to be important, with no prognostic relevance, with the exception of the rare *TRAF1*_*ALK* fusion transcript, which was shown to be associated with a poor outcome in a recent study (87). In children with ALK-positive ALCL, the prognosis also correlates with the ALK antibody titer and the copy number of the ALK fusion transcript in the blood at diagnosis (MDD: minimal disseminated disease) and after treatment (MRD: minimal residual disease) (189, 191–194). The significance of these parameters is unknown in adult patients.

In routine practice, FISH is required to diagnose DUSP22-rearranged ALK-negative ALCL, a molecularly distinct subgroup that probably merits being individualized (37). Interestingly, it is also characterized by the presence of hotspot mutations of *MSC* E116K in 35% of DUSP22-rearranged cases, a finding currently without clinical relevance (111). In the context of intestinal T-cell lymphomas, the identification of *SETD2* alterations strongly favors the diagnosis of MEITL and may be helpful in distinguishing difficult cases from EATL (18). These alterations (mutations and/or deletions) result in reduced H3K36 trimethylation, which can be detected by immunohistochemistry (195).

A number of genetic alterations may also predict the outcome of patients with a T- or NK-cell neoplasm, as observed in ENKTCL, with the poor prognosis of the MB subgroup (30), and in ATLL with CCR4 mutations or CCR7 alterations (146, 196–198). In AITL, the DNMT3A^R882X^ mutation may be associated with a poor prognosis and resistance to anthracyclines (199), a finding that could influence the management of these patients in the future. MYC expression/rearrangement or TP53 alterations are associated with a poor prognosis in various PTCL entities, especially ALK-positive ALCL (104, 105), ENKTCL (30) and MEITL (151), but without a significant impact on the management of these patients.

The diagnosis of ATLL is challenging for pathologists without knowledge of the HTLV-1 serology status. Morphological and immunophenotypic features may be confusing for ALK-negative ALCL, GATA3 PTCL-NOS, or even TFH-lymphomas, with an impact on the appropriate management of these patients. There is an unmet need for the development of HTLV-1 biomarkers applicable to FFPE samples in routine practice. TAX is not expressed in most ATLL tumors, whereas HBZ is the only viral transcript expressed during disease progression and could be a good candidate (50, 51). *In situ* hybridization was proposed to detect the *HBZ* gene in FFPE tissues in a single study, but there has thus far been no development of this technology in routine practice (200). More recently, targeted gene expression studies have been developed to measure expression of the *HBZ* transcript in routinely-fixed samples (181, 201).

Thus far, the detection of fusion transcripts has not been integrated into the routine diagnosis of PTCL due to the low prevalence of known fusions (10%) and limited accessibility to available technologies. Although RNAseq is the most exhaustive technology to detect fusion transcripts, several targeted RNA sequencing alternatives have been developed (ArcherFusionPlex^®^, Qiaseq RNA fusion XP^®^, and ld-RTPCR (202)), which can be implemented in a routine laboratory at a lower cost. Despite the current lack of clinical relevance of most fusion transcripts, the recent identification of rearrangements involving *JAK2* in systemic CD30-positive PTCL (119, 203), Bi-ALCL (124), in indolent clonal T-cell lymphoproliferative disorder of the gastrointestinal tract (153), and cutaneous T-cell lymphoma (204–208) opens the door to targeted therapies requiring the detection of such fusion transcripts. Furthermore, in addition to pathological features, the detection of certain transcripts may be of diagnostic value to support a diagnosis among several hypotheses. For example, *ICOS*_*CD28*, *ITK*_*SYK*, or *FYN*_*TRAF3IP2* fusions favor a diagnosis of PTCL, especially TFH-lymphoma in difficult cases, raising the possibility of the differential diagnosis from Hodgkin lymphoma or marginal zone lymphoma.

Recent studies on a limited number of cases have demonstrated the applicability of assessing circulating tumor DNA (ctDNA) by high-throughput sequencing for PTCL. In a comparison with matched tumors, ctDNA detected by HTS-sequencing of the TCR was detected for 78% of various PTCL entities (209). The detection of hotspot mutations in AITL (*RHOA* and *IDH2*) appears to be promising and sensitive, with 100% concordance between cell-free DNA and the tumors by NGS in one study (210) and a prevalence of 70% in another using allele-specific PCR (188). In ENKTCL, a concordance of 93.5% between ctDNA and tumor biopsy sequencing was observed, with a potential prognostic significance (211–213). Beyond the potential application for the detection of minimal residual disease during follow-up or at relapse, the detection of ctDNA may also be a promising tool to help for the diagnosis of difficult cases, especially those with limited tumor material, in combination with pathological analysis.

## 4 From molecular targets to personalized treatment: alternatives or additive therapeutic options to standard chemotherapy

The CHO(E)P-based regimen has been the standard of care for PTCL for many decades (214). To date, most alternative therapies have failed to demonstrate a better outcome and the prognosis of patients for most PTCLs is still poor (215, 216), even for stage I-II disease (217).

### 4.1 Frontline targeted therapies

A recent major change in frontline therapy is the use of brentuximab-vedotin (BV), in addition to CHP chemotherapy, for patients with CD30 positive PTCL. Approval for the use of BV by the US Food and Drug Administration (FDA) followed the ECHELON-2 study, which demonstrated a significant improvement in progression free survival (PFS) (median 48 months in the BV-CHP group versus 20.8 months in the CHOP group, p=0.0110), and a reduced risk of death in the BV-CHP arm, although the median overall survival (OS) was not reached (103). However, the subgroup analyses confirmed the benefit for ALCL patients receiving BV, but not for those with AITL. For PTCL-NOS, the potential benefit is unclear, probably due to the heterogeneity of the disease with respect to the percentage of CD30-positive cells (threshold ≥10% of cells by local review). The addition of BV to standard chemotherapy has also been shown to provide an improvement in event-free survival of children with ALK-positive ALCL (218). In addition, a retrospective pooled study showed a significant improvement of OS and PFS in ALK-positive ALCL with the use of CHOEP in frontline therapy compared to CHOP, independently of age (100). To date, there has been no comparison between CHOEP and BV-CHP in the frontline management of ALK-positive ALCL patients.

A second large trial compared the addition of romidepsin to CHOP versus CHOP alone in previously untreated PTCL patients (219). Although the results of the study were negative, as PFS did not statistically increase in the romidepsin CHOP group relative to the control arm, a trend towards longer PFS was observed for TFH-lymphoma patients, suggesting susceptibility of TFH-lymphomas to drugs targeting epigenetics. A phase 2 trial combining the oral form of 5-azacytidine to CHOP in the first line for 21 PTCL patients, including 17 with TFH-lymphoma, showed promising results, with an 88% complete response (CR) rate for TFH-lymphoma patients and 69% two-year PFS. However, these promising results, based on a limited number of patients, need to be confirmed in a larger series (220).

Among ENKTCL, the introduction of asparaginase has significantly improved the prognosis of patients (221, 222). Better efficacy and tolerance have been observed with the use of pegasparaginase relative to L-asparaginase (223, 224). Although there is no international consensus concerning the treatment sequence, it is generally accepted that frontline therapy should include at least pegylated-asparaginase and gemcitabine in association with various combination of other agents or strategies (including cisplatin/oxaliplatin, dexamethasone, methotrexate, and radiotherapy), depending on the staging of the lymphoma as localized or disseminated disease (223–226).

In ATLL, the characterization of a Treg/TH2 phenotype and polarization of the tumor cells led to the development of anti-CCR4 monoclonal antibodies (227). Although this targeted therapy is currently used for refractory/relapsed patients, a recent study showed better survival of aggressive transplant-ineligible ATLL using a polychemotherapy regimen containing mogalizumab in the first line (4-year OS of 46.3% versus 20.6%, p=0.033) (228). A previous study failed to demonstrate any benefit with the addition of mogalizumab in the first line for transplant-eligible patients (229). It is still unknown whether the use of mogamulizumab could be extended in the future to other PTCLs that express CCR4, in particular, GATA3-PTCL-NOS (164).

### 4.2 Promising therapeutic options for relapse/refractory PTCL patients

Several ALK inhibitors have been tested in refractory/relapsed ALK-positive ALCL patients, showing an improvement in PFS and long-term complete remission (230–234). However, there are no recommendations concerning the indication or duration of treatment.

The frequent alterations of chromatin modifiers among PTCLs has led to the development of therapies to regulate epigenetic programs. Although approved by the FDA, the use of romidepsin, pralatrexate, and belinostat did not show significant efficacy in several studies, probably due to the enrollment of patients with several PTCL entities, leading to a small sample size for each (219, 235). However, subgroup analyses showed a benefit for HDAC inhibitors for TFH-lymphomas (219, 236). Prospective studies are needed to confirm these promising results for TFH-lymphoma patients and to identify predictive biomarkers of response. Several studies using hypomethylating agents, such as 5’azacytidine, have also shown promising results in AITL, usually independently of the *TET2*, *DNMT3A*, and *IDH2* mutational status, although these studies had only small numbers of patients (237, 238). A phase 3 trial comparing the use of the oral form of the 5-azacytidine to investigator-choice treatment between gemcitabine, bendamustine, or romidepsin in relapsed/refractory THF-lymphoma patients was recently reported. The primary endpoint was PFS and was not met, likely due to the trial being underpowered. However, OS was longer for patients receiving 5-azacytidine, suggesting efficacy of the drug. The combination of oral 5-azacytidine and romidepsin has shown efficacy for frontline or refractory/relapsed PTCL patients, especially those with a TFH phenotype (239). The development of IDH2 Inhibitors in acute myeloid leukemia (240, 241) suggests their potential application in TFH-lymphomas with *IDH2* mutations.

In AITL, the identification of gene alterations enhancing the TCR pathway paved the way for the use of dasatinib, a PKC inhibitor, which showed efficacy *in vitro* and *in vivo* in a mouse *RHOA* G17V mutant *TET2* deleted model, as well as in a phase 1 trial for relapsed/refractory patients (71, 242).

The identification of structural alterations of PDL1 in ENKTCL led to studies to evaluate the use of immune checkpoint inhibitors, such as PD1 inhibitors, for refractory/relapsed patients (5, 132, 243). The response to these therapies may be predicted by characterization of the tumor immune microenvironment using gene expression profiling (Nanostring technology) or immunohistochemistry (anti-PDL1, anti-FOXP3, anti-CD68) (244). Surprisingly, although similar disruption of the 3’UTR of *PDL1* was also detected in ATLL, the use of PD1 inhibitors in this entity led to rapid progression of the disease for at least some patients (245).

Translocations involving *JAK2* leads to phosphorylation of the tyrosine kinase domain, subsequent constitutive activation, and downstream JAK/STAT pathway activation (246). This pathway is now targeted using JAK inhibitors in the clinic for myeloproliferative neoplasms and cancers with high pSTAT3 levels (247), such as ALK-negative ALCL, may be a good candidate for such targeted therapy, as suggested *in vivo* in a xenograft model (248). In a recent study, ruxolitinib showed some clinical activity on PTCLs, especially those with JAK or STAT mutations or activation (249).

Recently, the KIR3DL2 killer Immunoglobulin-like receptor was identified as a useful biomarker and therapeutic target among cutaneous T-cell lymphomas, including mycosis fungoides and Sezary syndrome (250, 251) and ATLL (252, 253). Its expression in other PTCL entities has been recently evaluated (254) and lacutamab, an anti KIR3DL2 antibody, is currently under investigation for KIR3DL2-positive PTCL (NCT04984837).

Given the limited efficacy of conventional chemotherapies, such as CHOP, for most PTCL patients, in the future, it may be worthwhile considering alternative treatment options that are personalized and directed according to the molecular characterization of the tumor (**Table 3**). However, whether the detection of actionable alterations will be clinically important for most PTCLs, which are still an unmet medical need for most, remains unknown.

**Table 3:** Relevant cytogenetic or molecular findings for the management of PTCL patients.

Entity	Diagnosis	Prognosis	Therapeutic relevance	Potential targeted therapies
**TFH-lymphoma**	- Mutations *RHOA* G17V, *IDH2* R172, - fusions transcript *ITK*_*SYK*	*DNMT3A* R882X	*ITK*_*SYK* *CTLA4_CD28* *FYN*_*TRAF3IP2*	Demethylating agents PI3K inhibitors SYK inhibitors CTLA4 inhibitors IkB inhibitors
**ALK-positive ALCL**	ALK expression (IHC), rearrangement (FISH), fusion transcript	MYC expression *TRAF1*_*ALK* fusion transcript		Brentuximab-vedotin ALK inhibitors JAK/STAT inhibitors
**ALK-negative ALCL**		FISH for: *-DUSP22* rearrangement *- TP63* rearrangement	JAK2 fusion transcripts pSTAT3	Brentuximab vedotin JAK/STAT inhibitors Kinase inhibitor
**ENKTCL (nasal type)**	EBV (EBER ISH)	MYC expression	PDL1 expression	Immune checkpoint inhibitors (pembrolizumab, nivolumab)
**HSTL**	Iso7q (FISH)		KIR3DL2 expression	Humanized KIR3DL2 antibodies (lacutamab) JAK/STAT inhibitors
**EATL**	Mutations *JAK1* p.G1097, *STAT3*			JAK/STAT inhibitors
**MEITL**	*SETD2* mutation/deletion	CD20 expression (favorable) *TP53* alterations, *MYC* expression		JAK/STAT inhibitors Wee1 inhibitor (adavosertib)
**Indolent NK-LP of the GI tract**	*STAT3 K563_C565del*			JAK-STAT inhibitors
**ATLL**	*HBZ* transcript	Aggressive: mutations of *CCR4* (frameshift), *TP53*, *IRF4* Indolent: *STAT3* mutations	KIR3DL2 expression	Humanized antibodies against CCR4 (mogamulizumab), KIR3DL2 (lacutamab)
**PTCL-NOS**		TH2 polarization DNMT3A mutations		Humanized antibodies against CCR4 (mogamulizumab)

FISH, fluorescence in situ hybridization; IHC, immunohistochemistry; ISH, *in situ* hybridization.

## 5 Conclusion

The emergence of innovative high-throughput technologies has led to a better understanding of the pathogenesis of most PTCL entities, highlighting their diversity in terms of their biology and clinical features. A large group of TFH-lymphoma patients has emerged with a unique lymphoma oncogenesis, for which the diagnosis takes advantage of robust molecular markers and for which the treatment may benefit from the emergence of novel therapies, such as those that target epigenetics. The ALCL category is still heterogenous due to its genetic diversity, which has prognostic relevance, but may now benefit from the introduction of BV targeting CD30. The recent description of the genetic landscape of PTCL offers the rationale for an association of targeted therapies, with or without conventional chemotherapy agents, in the future, although the efficient combination for each PTCL entity or molecular subgroups still needs to be identified.

## Author contributions

FD and PG wrote and supervised the manuscript. FL supervised the “oncogenic mechanisms” part, wrote and supervised the therapeutic part. All authors contributed to the article and approved the submitted version.
